# Association of meteorological factors and air NO_2_ and O_3_ concentrations with acute exacerbation of elderly chronic obstructive pulmonary disease

**DOI:** 10.1038/s41598-018-28532-5

**Published:** 2018-07-05

**Authors:** Ming-Tai Lin, Chew-Teng Kor, Chun-Chi Chang, Woei-Horng Chai, Maw-Soan Soon, Yi-Siang Ciou, Ie Bin Lian, Chia-Chu Chang

**Affiliations:** 1 Division of Chest Medicine, Department of Internal Medicine, Changhua Christian Hospital, Changhua 500, Taiwan; 2 Medical Research Center, Department of Internal Medicine, Changhua Christian Hospital, Changhua 500, Taiwan; 3 Institute of Statistics and Information Science, National Changhua University of Education, Changhua 500, Taiwan; 4 Division of Nephrology, Department of Internal Medicine, Changhua Christian Hospital, Changhua 500, Taiwan; 5 School of Medicine, Chung Shan Medical University, Taichung 404, Taiwan; 6 Department of Internal Medicine, Kuang Tien General Hospital, Taichung, Taiwan; 7 Department of Nutrition, Hungkuang University, Taichung, Taiwan

## Abstract

We studied the combined effect of air pollutant concentrations and meteorological factors [e.g., temperature and atmospheric pressure (AP)] on the acute exacerbation of coronary obstructive pulmonary disease (COPD) in 277 older patients with COPD (240 men and 37 women; average age, 75.3 ± 9.3 years). Average air pollutant concentrations, AP, temperature, and relative humidity corresponding to each of the 7 days before the date of hospitalisation were identified as the case and the two other weekly averages, 4 and 8 weeks prior to admission, were considered the controls. During the warming-up season, COPD exacerbation more likely occurred on days of temperature increase or AP decrease than on other days. Increments in CO, NO_2_ and O_3_ concentrations were significantly associated with 5%, 11% and 4% increases in COPD exacerbation risks, respectively. During the cooling-down season, increments in PM_10_ concentrations were significant risk factors; the exacerbation likely occurred on days of temperature decreases than on other days. Air pollution with increased NO_2_, CO, O_3_ and PM_10_ concentrations and continual temperature changes (colder during cooling-down seasons or hotter during warning-up seasons) were associated with acute exacerbation of COPD in older patients.

## Introduction

Chronic obstructive lung disease is the seventh leading cause of mortality in Taiwan^1^ and the third leading cause of mortality in the United States^2^. The prevalence and mortality rate of chronic obstructive pulmonary disease (COPD) may increase in the next decade. The World Health Organization (WHO)^3^ has estimated that COPD will be the third leading cause of global mortality by 2020. Hospitalisation for acute exacerbated COPD (AECOPD) is recognised as a major event in the natural history of COPD because of its negative effect on lung function, survival, risk of readmission, and quality of life^4,5^. The admission fee due to AECOPD presents an excessive economic load. COPD exacerbation is the major direct and indirect cost driver^6^. Early identification and effective COPD management are crucial for reducing COPD burden^7^. Discovering potential factors contributing to exacerbations among patients with COPD would be helpful for adopting preventive measures and reducing the risk of death. Many precipitating factors can lead to COPD exacerbation, including older age, lower body mass index, poor lung function, and a higher frequency of exacerbations and comorbidities^8^. Exposure to some environmental factors can also cause the exacerbation, such as air pollutants, occupational hazards, and infections^9^; however, their effects remain poorly understood. Among these is air temperature. AECOPD is triggered by bacterial and viral infection, air pollution, and other unknown factors. However, most studies have investigated the association between AECOPD and the effect of climate change in cold weather. Studies on the effects of seasonal variations and temperature on AECOPD are lacking. The anticipated effects of climate change also include increases in temperature variability and extreme cold weather conditions. Excessively cold temperatures have been linked to increases in the mortality and morbidity of those with COPD. In a study of individuals aged older than 65 years in Michigan, USA, those with COPD had a 19% increased risk of dying on cold days^10^. In a New Zealand study, the death rate was 18% higher in winter compared with other seasons and 31% of additional deaths were attributable to respiratory disease^11^. A large case-crossover study in Taiwan reported a 0.8% increase in the risk of COPD exacerbations for every 1 °C decrease in the mean daily temperature in cold weather^7^. Unlike air pollution, which has a monotonic, linear dose–response relationship (higher pollution is associated with higher mortality), the associations observed with temperature are often nonlinear, particularly in climates where physiologically stressful temperatures occur on either side (colder or hotter) of a zone of relatively comfortable temperatures^12,13^.

This study investigated the association between AECOPD and the effects of climate change during different seasons, including changes in temperature, atmospheric pressure (AP), and fine particulate matter (PM_2.5_) concentration.

## Methods

### Study population and data source

This 5-year case-crossover study was conducted with patients enrolled at the Changhua Christian Hospital (CCH), the largest medical centre receiving patients primarily from Changhua County in Taiwan. All methods were performed in accordance with the relevant guidelines and regulations. The study enrolled 277 patients with COPD who had all experienced an exacerbation of their condition and were hospitalised at the CCH during 2011–2015.

The inclusion criteria were (1) admission to CCH due to AECOPD, (2) age of 65–85 years, and (3) AECOP-related visitation with a principal diagnosis according to International Classification of Diseases (ICD) 9 codes (491.2, 496, and 491.21). To confirm the reliability of the ICD-9 codes, all patients underwent a pulmonary function test for the ratio of forced expiratory volume in 1 second (FEV1)-to-forced vital capacity (FVC); only the patients with an FEV1/FVC ratio of <70% were included. The exclusion criteria were (1) age of <65 years and (2) a diagnosis of lung cancer. The exacerbation criterion were an acute worsening of respiratory symptoms resulting in administration of additional therapy and requiring hospital admission including a situational awareness bridge display plus antibiotics or corticosteroids.

This study was approved by the Institutional Review Board of the CCH (protocol no: CCH: P201507-45).

### Meteorological and air pollution data

**Figure 1:**
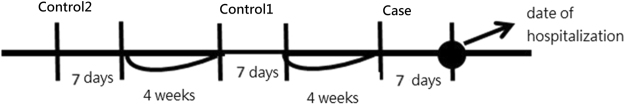
Case-crossover study design: one case versus two controls.

According to their address, each patient’s area of residence was linked to the Air Quality Monitoring Network (AQMN) established by the Taiwan Environmental Protection Administration (EPA) and the Central Weather Bureau (CWB) data bank, which have monitoring stations distributed throughout the area to monitor air quality and meteorological factors on an hourly basis. The following case-crossover design was applied to each patient (Fig. 1): The averaged measurements of air pollutant concentrations, AP, temperature, and relative humidity (RH) corresponding to each of the 7 days before the date of hospitalisation were included as the case and the two other weekly averages, at 4 weeks and 8 weeks before admission, were employed as the controls.

**Figure 2:**
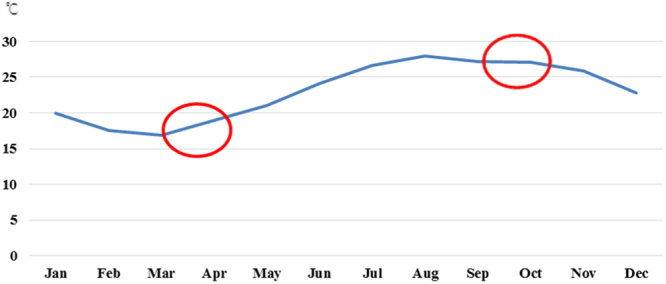
Monthly temperatures averaged over 2010–2015.

Meteorological factors and pollutant levels are closely related to change in seasons^14–16^. Furthermore, that a separate analysis for cooling-down and warming-up seasons is crucial because factors, such as temperature, may affect each patient differently. Figure 2 provides a plot of the averaged monthly temperature trend in Changhua over the study period. April and October are the two evident turning points—the temperature increased from April to August, remained stable through September, and decreased drastically from October to March of the following year. We believe that the change in temperature had a more crucial effect on COPD exacerbation than the average temperature; therefore, we divided a year into warming-up season and cooling-down season with periods from April to September and from October to March of the next year, respectively.

Regarding the AQMN, established in September 1993, 77 monitoring stations are currently established in 75 townships and precincts in Taiwan. Based on each patient’s address, the closest monitor station was selected; consequently, data from 13 monitor stations were used. We archived the complete monitoring data for air pollutants, including NO_2_, CO, SO_2_, PM_10_, PM_2.5_, and O_3_, as well as daily temperature and RH from the AQMN database. Concentrations of each pollutant were continuously measured, and reported hourly, through nondispersive infrared absorption for CO, chemiluminescence for NO_2_, ultraviolet absorption for O_3_, ultraviolet fluorescence for SO_2_, and b-gage for PM_2.5_. A daily (24-h) average concentration was calculated when at least 13 valid hourly values were available with no more than six successive hourly values missing. The 8-h averaged concentration was calculated when at least six valid hourly values were available. The exposure was calculated on the basis of the average 24-h daily records for NO_2_, CO, SO_2_, PM_10_, and PM_2.5_ concentrations, and the 8-h daily records for O_3_ concentrations (10:00 AM to 6:00 PM)^17^. The hourly weather records, including temperature and RH, were also obtained from the AQMN, whereas the atmosphere pressure was obtained from the Taiwan EPA and CWB. Before calculating the daily average, the abnormal maximal and minimal measures were double-checked to avoid the input of errors.

### Statistical analysis

Data were expressed as percentages and mean ± standard deviations (SDs) for categorical or continuous variables, respectively. The univariate comparison for categorical variables was determined using the chi-square test and continuous variables were determined using a Student’s *t* test. To overcome the dependency between the case and control, a conditional multiple logistic regression model was used to estimate the odds ratios (ORs) and their 95% confidence intervals (CIs) for risk factors. Each patient was considered a stratum with one case and two controls. The advantage of the case-crossover design is that variables, such as gender or chronic diseases, can be ignored because they are invariant within each stratum (patient). The adjusted variables in the conditional multiple logistic regression model were the metrological variables, including RH, AP, and ambient temperature (TX), and the concentrations of the six air pollutants. Furthermore, test of interaction in conditional logistic models to determine the association between meteorological factors, air pollution and AECOPD across warming-up and cooling-down seasons. Indicating statistically significant interaction effects with P_interaction_ < 0.05, it means that meteorological factors, air pollution showed differential effects across warming-up and cooling-down seasons. Each variable was measured as the average across a 7-day period, as presented in Fig. 1. A two-tailed P of <0.05 was considered to be statistically significant. All analyses were performed using the statistical software package SAS for Windows (version 9.3; SAS Institute Inc., Cary, NC, USA).

## Results

### Demographics

In total, 277 patients with COPD (240 men and 37 women) were recruited in this study, and the mean age of participants was 75.3 ± 9.3 years. Of them, 137 had exacerbations in the cooling-down season, whereas the remaining 140 experiencing exacerbations in the warming-up season. Table 1 represents the patient characteristics between the cooling-down and warming-up seasons. No significant differences in age, gender, and history of comorbidities, including hypertension, diabetes mellitus (DM), coronary artery disease (CAD), and congestive heart failure (CHF), were noted (all P > 0.05). However, meteorological factors and air pollution were significantly different between warming-up and cooling-down seasons, except SO_2_ and O_3_. At least one of the comorbidities was noted in 171 patients (61.7%). Table 2 lists the averaged diurnal measurement (±SD) of meteorological factors and air pollutants for the cases and controls in the respective seasons. The univariate comparison between the cases and controls indicated no significant difference in RH and AP; however, a reversal in differences in the two seasons were observed. In the cooling-down season, patients lived in a colder climate than the controls (19.36 °C vs 21.51 °C), whereas in the warming-up season, the patients lived in a hotter climate than the controls (27.02 °C vs 23.74 °C). The cooling-down season had severer air pollution than the warming-up season, except in case of O_3_.

**Table 1:** Demographic and clinical characteristics of patients with COPD, stratified by the season of exacerbation.

N	Warming-up	Cooling-down	P-value
	140	137	
Gender (M:F)	119:21	121:16	0.525
Age	75.07 ± 9.02	75.34 ± 9.57	0.524
Hypertension	60 (24.8%)	69 (28.5%)	0.258
DM	29 (12.0%)	20 (8.3%)	0.240
CAD	27 (11.2%)	16 (4.1%)	0.114
CHF	11 (4.6%)	10 (6.6%)	0.959
RH (%)	76.09 ± 6.39	73.84 ± 6.45	0.004
TX (°C)	27.02 ± 2.78	19.36 ± 3.64	<0.001
AP (hPa)	992.46 ± 33.08	1003.17 ± 29.12	0.005
CO (10 ppb)	35.56 ± 11.98	51.67 ± 11.26	<0.001
PM_10_ (ug/m3)	47.3 ± 15.79	61.33 ± 16.04	<0.001
PM_2.5_ (ug/ m3)	26.82 ± 10.61	36.09 ± 10.06	<0.001
NO_2_ (ppb)	12.44 ± 4.77	18.16 ± 4.19	<0.001
SO_2_ (ppb)	3.52 ± 1.02	3.7 ± 0.96	0.119
O_3_ (ppb)	29.24 ± 8.11	27.62 ± 7.75	0.092

**Table 2:** Univariate comparison of the meteorological and air pollutant average diurnal measures (±standard deviation) between cases and controls, for the cooling-down and warming-up seasons, respectively.

		RH (%)	TX (°C)	AP (hPa)	CO (10 ppb)	PM_10_ (ug/m^3^)	PM_2.5_ (ug/m^3^)	NO_2_ (ppb)	SO_2_ (ppb)	O_3_ (ppb)
**Cooling–down season**										
(N = 137)	Case	73.84 ± 6.45	19.36 ± 3.64	1003.17 ± 29.12	51.67 ± 11.26	61.33 ± 16.04	36.09 ± 10.06	18.16 ± 4.19	3.7 ± 0.96	27.62 ± 7.75
	Control	73.26 ± 7.02	21.51 ± 5.03	1001.52 ± 29.21	48.05 ± 13.15	60.31 ± 16.81	35.94 ± 10.75	17.10 ± 5.01	3.85 ± 1.03	28.66 ± 8.27
	P-value	0.406	<0.0001	0.589	0.006	0.552	0.889	0.033	0.142	0.213
**Warming–up season**										
(N = 140)	Case	76.09 ± 6.39	27.02 ± 2.78	992.46 ± 33.08	35.56 ± 11.98	47.3 ± 15.79	26.82 ± 10.61	12.44 ± 4.77	3.52 ± 1.02	29.24 ± 8.11
	Control	76.34 ± 6.42	23.74 ± 4.92	995.6 ± 34	41.52 ± 15.11	55.61 ± 20.43	31.99 ± 12.81	14.66 ± 5.66	3.57 ± 1.01	28.56 ± 6.9
	P-value	0.711	<0.0001	0.365	<0.0001	<0.0001	<0.0001	<0.0001	0.624	0.371

RH: Relative humidity, AP: atmospheric pressure, TX: ambient temperature.

### Associations among meteorological factors, air pollutants, and COPD exacerbation

Because of the strong correlations among the six air pollutants, we fitted six models for both seasons, with all metrological variables and a single air pollutant, namely Model 1 on RH + AP + TX + CO, and Model 2 on RH + AP + TX + PM_10_.

**Figure 3:**
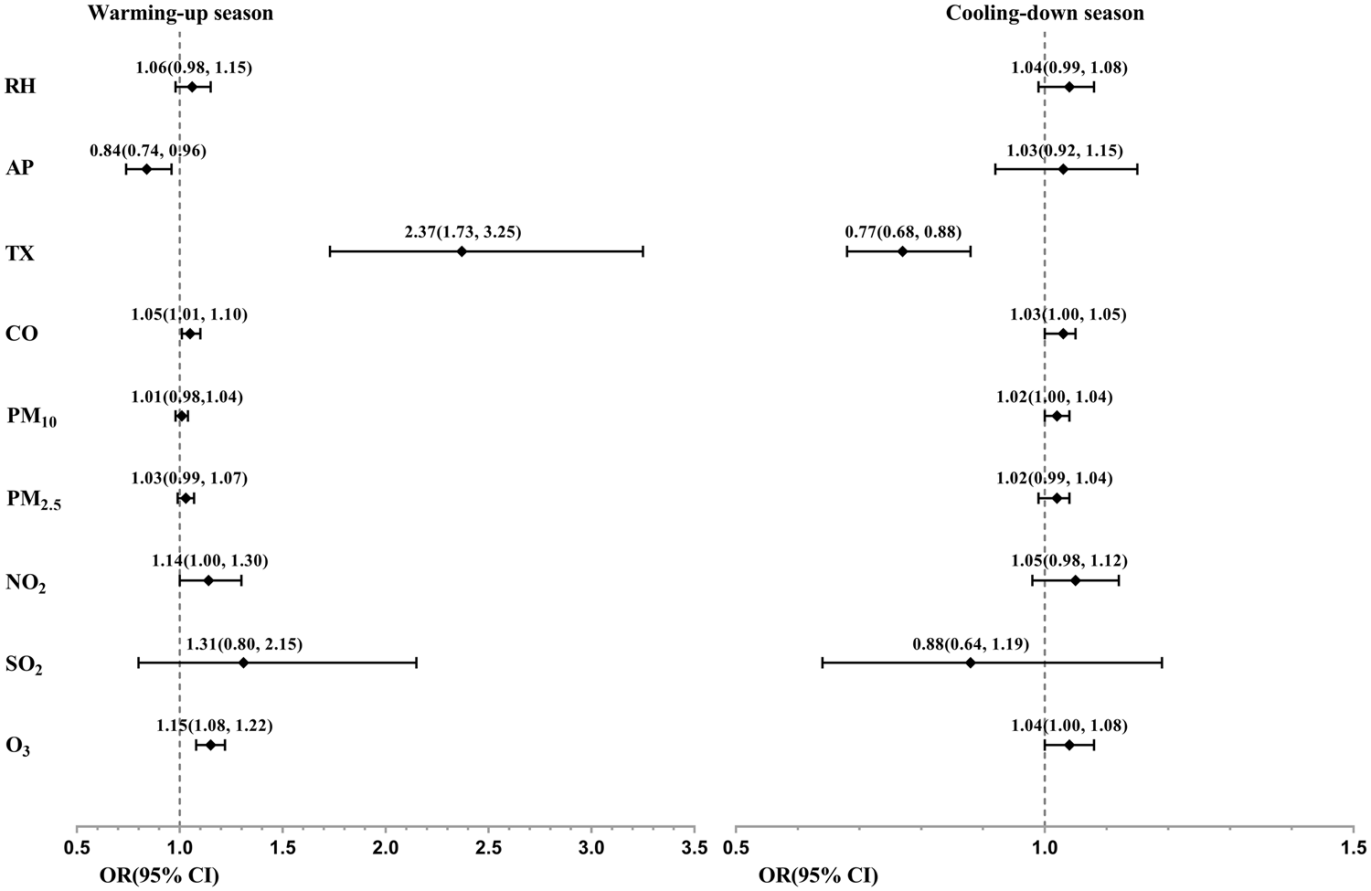
Forest plot of the ORs and 95% CIs of risk factors of exacerbation, for all patients with COPD stratified by season.

Tables 3 and 4 list the results from the conditional multiple logistic regressions using all patients with COPD separated into warming-up and cooling-down seasons. The corresponding ORs and 95% CIs for each risk factor are illustrated by the forest plot in Fig. 3. In the warming-up season, Model 1 in Table 3 revealed an increase in TX by 1 °C and decrease in AP by 1 hPa, with 95% CIs of ORs of 1.73−3.25 and 0.74−0.96, respectively, and corresponding P of <0.001 and 0.012, respectively. Similar results were observed in the other models. For air pollutants factors, Model 1, 4 and 6 revealed that increases in CO concentrations by 10 part per billion (ppb), NO_2_ and O_3_ concentrations by 1ppb are significant risk factors for COPD exacerbation, with 95% CIs of ORs of 1.01−1.10, 1.00−1.30 and 1.08−1.22, respectively, and corresponding P of 0.024, 0.044 and <0.001, respectively. In the cooling-down season (Table 4), increments in PM_10_ concentrations were significant risk factors, with 95% CIs of ORs of 1.00−0.04, and corresponding P of 0.017. However, TX had the opposite effect, with exacerbation more likely to occur in days with a decrease in TX than others, with the 95% CI of OR of 0.68−0.88 and P of <0.001. All five other models achieved similar results. Moreover, test of interaction revealed that AP (P_interaction_ = 0.024), TX, CO, PM_10_, PM_2.5_, NO_2_ (all P_interaction_ < 0.001) and O_3_ (P_interaction_ = 0.034) had significant interaction effects across warming-up and cooling-down seasons. However, RH with P_interaction_ = 0.580 and SO_2_ with P_interaction_ = 0.342 did not observed a differential effects between these two seasons.

**Table 3:** Conditional multiple logistic regressions for all patients with chronic obstructive pulmonary disease (COPD) in the warming-up season, with odds ratio (ORs) and 95% confidence intervals (CIs) for relative humidity (RH), atmospheric pressure (AP), ambient temperature (TX), along with single air pollutants.

Warming-up season	Model 1		Model 2		Model 3		Model 4		Model 5		Model 6	
	OR (95% CI)	P-value	OR (95% CI)	P-value	OR (95% CI)	P-value	OR (95% CI)	P-value	OR (95% CI)	P-value	OR (95% CI)	P-value
RH	1.06(0.98–1.15)	0.133	1.07(0.98–1.16)	0.127	1.06(0.98–1.15)	0.123	1.07(0.99–1.16)	0.090	1.06(0.98–1.15)	0.116	1.2(1.09–1.33)	<0.001
AP	0.84(0.74–0.96)	0.012	0.85(0.75–0.97)	0.015	0.85(0.75–0.97)	0.015	0.85(0.74–0.97)	0.014	0.83(0.73–0.96)	0.010	0.90(0.78–1.03)	0.134
TX	2.37(1.73–3.25)	<0.001	2.05(1.56–2.69)	<0.001	2.15(1.62–2.85)	<0.001	2.27(1.68–3.06)	<0.001	1.96(1.53–2.53)	<0.001	3.18(2.11–4.77)	<0.001
CO	1.05(1.01–1.10)	0.024										
PM_10_			1.01(0.98–1.04)	0.452								
PM_2.5_					1.03(0.99–1.07)	0.183						
NO_2_							1.14(1.00–1.30)	0.044				
SO_2_									1.31(0.80–2.15)	0.288		
O_3_											1.15(1.08–1.22)	<0.001

**Table 4:** Conditional multiple logistic regressions for all patients with chronic obstructive pulmonary disease (COPD) in the cooling-down season, with odds ratios (ORs) and 95% confidence intervals (CIs) of relative humidity (RH), atmospheric pressure (AP), ambient temperature (TX), along with single air pollutants.

Cooling-down season	Model 1		Model 2		Model 3		Model 4		Model 5		Model 6	
	OR (95% CI)	P-value	OR (95% CI)	P-value	OR (95% CI)	P-value	OR (95% CI)	P-value	OR (95% CI)	P-value	OR (95% CI)	P-value
RH	1.04(0.99–1.08)	0.133	1.06(1.01–1.11)	0.022	1.04(0.99–1.09)	0.081	1.04(0.99–1.09)	0.099	1.03(0.98–1.08)	0.202	1.07(1.01–1.13)	0.022
AP	1.03(0.92–1.15)	0.587	1.03(0.92–1.15)	0.652	1.04(0.93–1.16)	0.514	1.04(0.93–1.16)	0.526	1.06(0.95–1.18)	0.327	1.05(0.94–1.18)	0.376
TX	0.77(0.68–0.88)	<0.001	0.74(0.65–0.84)	<0.001	0.76(0.67–0.86)	<0.001	0.77(0.68–0.87)	<0.001	0.78(0.68–0.88)	<0.001	0.74(0.65–0.85)	<0.001
CO	1.03(1.00–1.05)	0.072										
PM_10_			1.02(1.00–1.04)	0.017								
PM_2.5_					1.02(0.99–1.04)	0.236						
NO_2_							1.05(0.98–1.12)	0.186				
SO_2_									0.88(0.64–1.19)	0.396		
O_3_											1.04(1.00–1.08)	0.055

### Effects of meteorological factors and air pollutants on exacerbation rates in stratified subgroup factors

**Figure 4:**
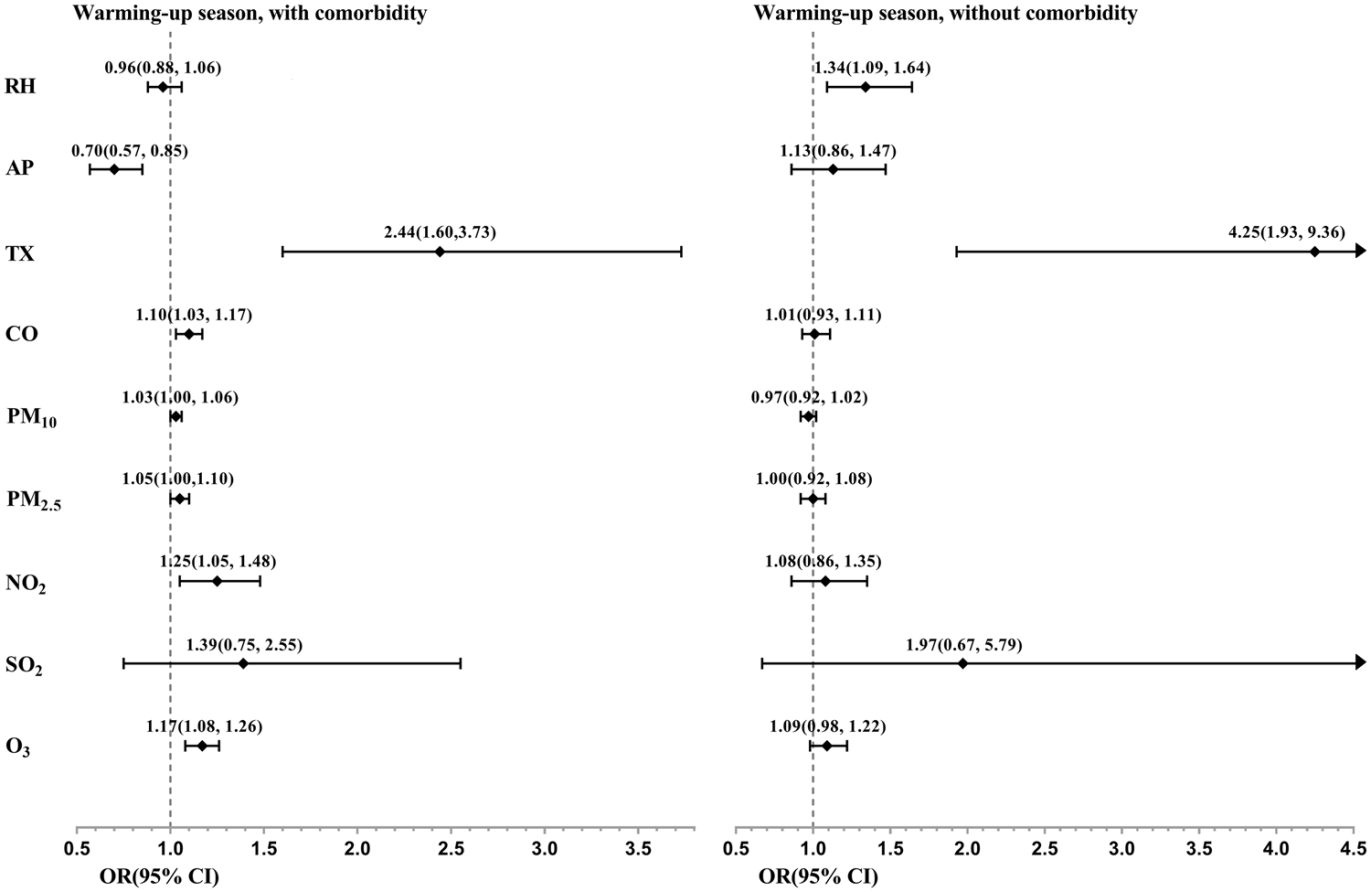
Forest plot of ORs and 95% CIs of risk factors of exacerbation, stratified by comorbid condition (with at least one of the followings hypertension, diabetes, CAD or CHF, vs. those without) in the warming-up season.

To further determine if different risk factors affected more vulnerable populations, we stratified the patients with COPD into two groups: those with at least hypertension, diabetes, CAD or CHF and those without any of these comorbidities. For those with comorbidities, the results were similar to the general population of patients with COPD, but with more significant risk factors and severer effects. In the warming-up season, an increase in TX, a decrease in AP, and an increase in CO, NO_2_, PM_2.5_, and O_3_ concentrations were significant risk factors for COPD exacerbation (Fig. 4). The ORs resulting from an increase in TX by 1 °C and a decrease in AP by 1 hPa were approximately 2.44, and 1.42 (inverse of 0.70), respectively. The OR for increases in CO concentration by 10 ppb was 1.10; for 1-ppb increases in NO_2_ and O_3_ concentration, it was 1.25 and 1.17, respectively, and it was 1.05 for 1-μg/m^3^ increases in PM_2.5_ concentration, respectively. By contrast, for those without specific comorbidities in the warming-up season, none of the aforementioned factors were significant, except TX (Fig. 4).

**Figure 5:**
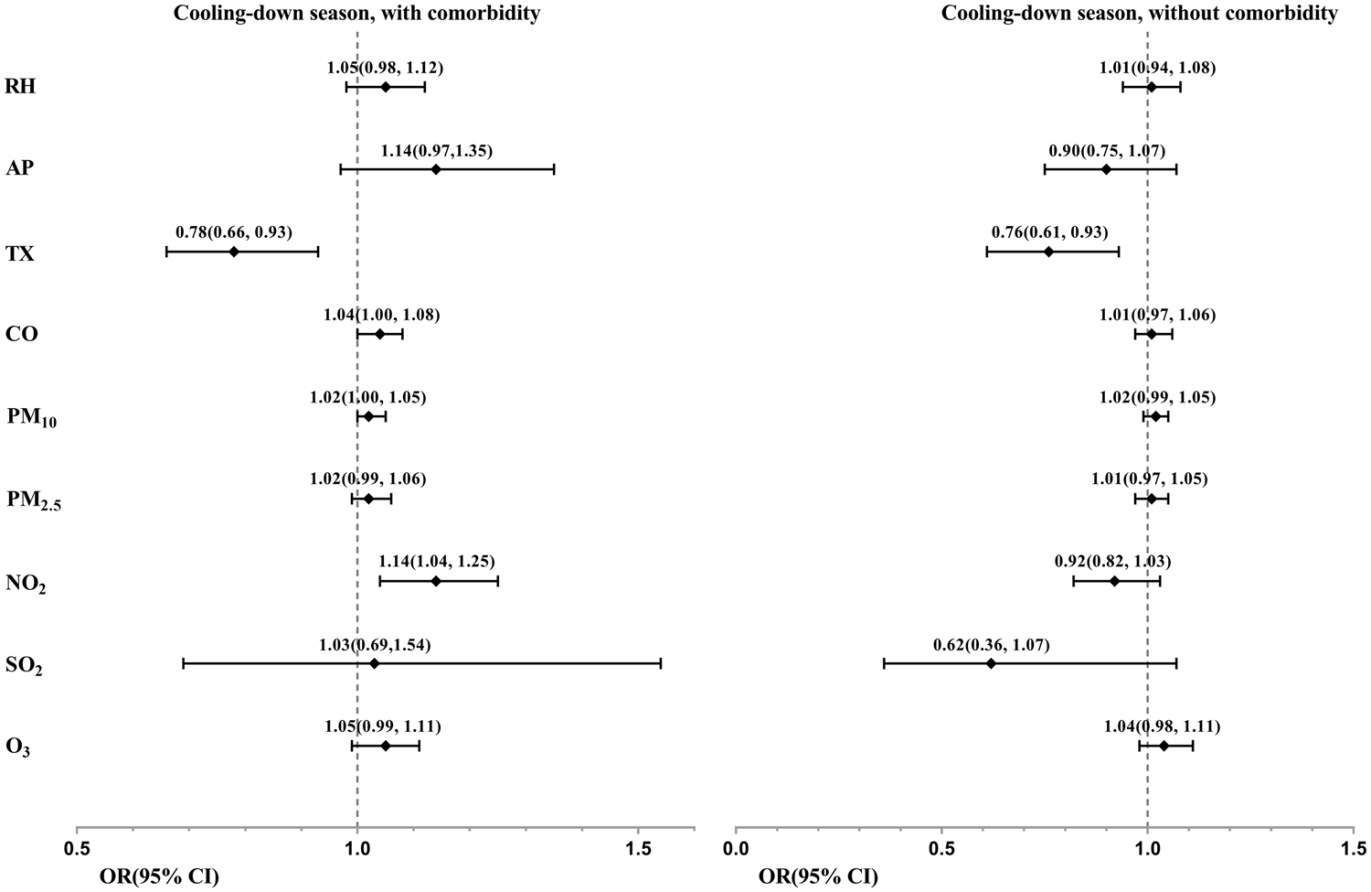
Forest plot of the ORs and 95% CIs of risk factors of COPD exacerbation, stratified by comorbid condition (with at least one of the following: hypertension, diabetes, CAD or CHF, vs without) in cooling-down season. The authors declare no competing interests, both financial and non-financial interests.

For patients with comorbidities in the cooling-down season, a decrease in TX, increase in NO_2_, and PM_10_ concentrations were significant risk factors for exacerbated COPD (Fig. 5). The OR for a decrease in TX by 1 °C, was approximately 1.28 (inverse of 0.78). The OR for an increase in NO_2_ concentration by 1ppb, and PM_10_ concentration by 1 μg/m^3^ were 1.14, and 1.02, respectively. By contrast, for patients without specific comorbidities in the cooling-down season, none of the above factors were significant, except TX (Fig. 5).

## Discussion

This study examined the relationship among climate, air pollutants, and AECOPD using a case-crossover design through the analysis of 277 patients with AECOPD from central Taiwan admitted to the CCH. We identified temperature as a potential risk factor for AECOPD, with opposite effects in different seasons. An increase in temperature in the warming-up season and a decrease in temperature in the cooling-down season were both associated with an increased risk of AECOPD. The reversal in TX-associated COPD mortality between hot and cold seasons has also been observed in other studies^18^. For example, two studies in the United States revealed that in hot weather days, an increase in temperature is associated with an increase of COPD hospitalization^19^ or a decrease in the survival rate of older people with chronic diseases^20^, whereas another study demonstrated that a decrease in temperature is associated with an increase in COPD exacerbation. Our study revealed that air pollutants, such as increased CO, NO_2_ and O_3_ concentrations, contributed to AECOPD in the warming-up season. Other studies have also indicated that the combination of heat stress and a high concentration of ambient air pollutants, including NO_2_ and O_3_, could cause inflammation of the bronchial mucosa as well as a reduction in the bronchoconstriction threshold, increasing the risk of acute injury to the lung tissue^21^. NO_2_ and O_3_ exposure have been demonstrated to trigger an inflammatory response, including *in vitro* and *in vivo* increases in IL-8 concentrations^22–24^. IL-8 is a potent neutrophil chemoattractant^25^, and neutrophil elastase is a powerful stimulant of mucin production^26^. Increased systemic and sputum IL-8 concentrations have been associated with COPD exacerbation^27,28^. C-reactive protein and fibrinogen, two other biomarkers for COPD, had also been revealed to be associated with an increase in ambient NO_2_ concentration^28^. Here, NO_2_ had significant effects on COPD exacerbation in both seasons, especially in comorbid patients, whereas the effects of O_3_ were significant only in the warming-up season, may be because O_3_ was the only pollutant with a higher concentration in the warming-up season than in cooling-down season (Table 2). Another explanation could be that the warming-up season in Changhua is not as hot as other regions at nightfall; thus, most people spend more time engaging in outdoor activities, thereby increasing their exposure to O_3_. Although NO_2_ did not seem to have a larger OR than CO or O_3_, it had considerably higher variances. However, in terms of concentration, NO_2_ molecules are smaller than O_3_ in both average and SD, which means that a 1-ppb change in NO_2_ is more severe than the same in O_3_; therefore, we did not directly compare the effects among different pollutants.

In our study, we revealed that a decrease in AP contributed to acute exacerbation in the warming-up season. This has not been analysed in other studies^7,20,21^. In other words, a typhoon period may induce AECOPD because during summer in Taiwan, typhoons are more frequent with low AP circulation. The reasons that low AP contribute to acute COPD exacerbation require further investigation.

Several studies have also addressed the interaction effect between temperature and air pollution on health issues such as mortality rates^29–32^. Among these studies, Hansel *et al*.^29^ specifically focused on COPD exacerbation and revealed that both extremely hot and extremely cold temperatures increased COPD morbidity. However, the AP was considered in this study. Zhu *et al*.^33^ and Ji *et al*.^34^ stated that increase in PM_10_ and PM_2.5_ concentration increased COPD hospital admissions; however, PM_10_ in cooling down season and PM_2.5_ for comorbid patient in warming-up season was observed in our study.

Comorbidities, such as myocardial infarction, DM, hypertension, and dyslipidaemia were associated with health issues including hospital readmissions for COPD^35^, as well as systemic inflammation that characterises the disease of COPD in previous studies^36,37^. In our study, the most common comorbidities were hypertension (46.6%), DM (17.7%), CAD (15.5%), and CHF (8.6%). To our knowledge, no previous studies have identified a relationship between climate change and COPD with comorbidity-induced AECOPD requiring hospital admission^7,35–38^. Our study analysed patients with COPD with at least one comorbidity (hypertension, DM, CAD, or CHF) are vulnerable to TX changes and exposure to air pollutants.

In this study, we defined the warming-up and cooling-down season based on changing temperature trends, rather than the average temperature of the months, because the variation or the increasing and decreasing trend of temperature trends may have a more crucial effect than average temperature on COPD exacerbation. For example, both January in the cooling-down season and May in the warming-up season could have the same average temperature of 20 °C; however, the 20 °C in January, a decrease from 22 °C in the previous month, is likely to be perceived as colder than the 20 °C in May, an increase from 18 °C in the previous month. By contrast, 26 °C in July, an increase from 24 °C in the previous month, is likely to be perceived as hotter than 26 °C November, a decrease from 28 °C in the previous month.

Our study has several limitations. First, relatively few cases of COPD exacerbation were noted in the warm season, which was similar to the study of Tseng^7^ performed in Taiwan, probably because Taiwan is a subtropical region with higher hospitalisation and morbidity rates in winter. We could not divide typhoon and nontyphoon periods for comparison in the warm season. Our study did determine that a decrease in AP was a significant risk factor for COPD exacerbation in the warm season during relatively low AP in typhoon periods in Taiwan. Second, although many confounders, such as individual lifestyles (e.g., socioeconomic status, health behaviours, nutritional status, and the use status of healthcare facilities), were adequately controlled using the case-crossover design, some relevant information, such as indoor temperature, was unavailable. The meteorological and air pollutant data provided by the EPA and CWB monitoring stations may not reflect the actual exposure of temperature and air pollutant data in patients with COPD, but we linked patients’ addresses to the nearest monitoring stations. Finally, all patients were recruited from one hospital and some selection bias should be considered.

## Conclusions

Air pollution, with an increase in PM_10_, NO_2_, CO, and O_3_ concentrations, as well as a continual change in temperature (i.e. getting colder in the cooling-down season or hotter in the warming-up season) were associated with AECOPD in older patients. Patients with comorbidities can be more vulnerable to the adverse effects of climate change and exposure to air pollutants. During the typhoon season in Taiwan, patients with COPD should take precautions to prevent COPD exacerbation.
